# Intestinal obstruction and ischemia by necrotic annular Meckel’s diverticulum: Case report and review of the literature

**DOI:** 10.1016/j.ijscr.2021.105897

**Published:** 2021-04-20

**Authors:** Giuseppe Evola, Sebastiano Caramma, Giovambattista Caruso, Riccardo Schillaci, Carlo Reina, Giuseppe Angelo Reina

**Affiliations:** a General and Emergency Surgery Department, Garibaldi Hospital, Catania, Italy; b General Surgery Department, San Salvatore Hospital, Paternò, Catania, Italy

**Keywords:** Meckel’s diverticulum, Intestinal obstruction, Intestinal ischemia, Management, Emergency surgery, Case report

## Abstract

**Introduction and importance:**

Meckel’s diverticulum (MD) is a vestigial remnant of the omphalomesenteric duct, representing the most common congenital malformation of the gastrointestinal tract. Diagnosis of MD is a challenge because of its rarity and frequent asymptomaticity. Radiological exams generally aren’t useful for its diagnosis. Intestinal obstruction represents the most common complication of MD in adults. Surgery is the appropriate treatment of complicated MD.

**Case presentation:**

A 70-year-old Caucasian male was admitted to the Emergency Department with a two-day history of abdominal pain associated with inability to pass gas or stool, nausea and vomiting. Physical examination revealed abdominal distention and abdominal pain without Blumberg’s sign. Abdominal contrast-enhanced computed tomography (CECT) showed small bowel obstruction caused by suspected MD. Laboratory tests reported high serum levels of glycemia, LDH, C-reactive protein and leukocytosis. After diagnosis of intestinal obstruction, the patient underwent exploratory laparotomy: a segmental resection of ischemic distal ileum bearing a necrotic MD was performed. The postoperative course of patient was uneventful.

**Clinical discussion:**

MD is found in 2%–4% of the population in large autopsy and surgical series. MD is mostly asymptomatic and incidentally discovered if not complicated; a debate exist about management of asymptomatic MD. Surgery represents the definitive treatment of complicated MD.

**Conclusion:**

MD is a true diverticulum rarely discovered in adults. Diagnosis of MD is difficult even with the help of radiological exams. Although surgical resection represents the correct treatment of symptomatic MD, nowadays there is no consensus on the optimal treatment of asymptomatic and incidentally discovered MD.

## Highlights

• Meckel’s diverticulum (MD) is the most common congenital malformation of the gastrointestinal tract.
• Annular MD is an extremely rare cause of intestinal obstruction and ischemia in adults.
• Preoperative diagnosis of MD is a challenge because of its rarity and the absence of specific radiological findings and symptoms.
• Surgery represents the appropriate treatment of complicated MD.

## 1 Introduction

The first description of Meckel’s diverticulum (MD) was in 1598 by Hildanus, but its name derived from the German anatomist Meckel who described the embryological and pathological features in 1809 [1]. MD is a vestigial remnant of the omphalomesenteric duct, representing the most common congenital malformation of the gastrointestinal tract [2]. It is a true intestinal diverticulum located on the antimesenteric border of the small bowel. Diagnosis of MD is a challenge because of its rarity and frequent asymptomaticity, the low diagnostic value of radiological exams and the absence of specific symptoms and signs. MD is usually asymptomatic being found incidentally during small bowel contrast study or abdominal surgery performed for unrelated conditions or until complications originating from the same diverticulum. When MD is symptomatic it may cause lower gastrointestinal hemorrhage, intestinal obstruction and diverticulitis with or without perforation. Intestinal obstruction is the most common clinical presentation of MD in adults. Surgery represents a diagnostic method and the correct treatment of a complicated MD, although a debate exists regarding the appropriate management of a silent MD incidentally discovered during surgery. A case of necrotic annular MD causing intestinal obstruction and ischemia is presented with review of the literature in accordance with SCARE 2020 criteria [3]. The purpose of this case report is to remember that annular MD is a extremely rare cause of intestinal obstruction that requires emergency surgery.

## 2 Presentation of case

**Fig. 1:**
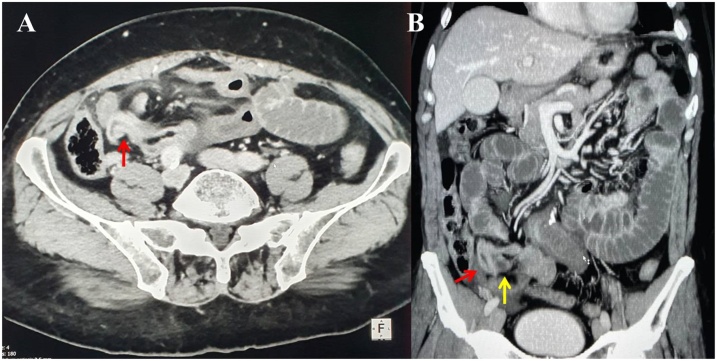
A,B. Preoperative abdominal CECT showing MD (red arrow) and strangulated distal ileal loop (yellow arrow). A transverse view, B coronal view.

**Fig. 2:**
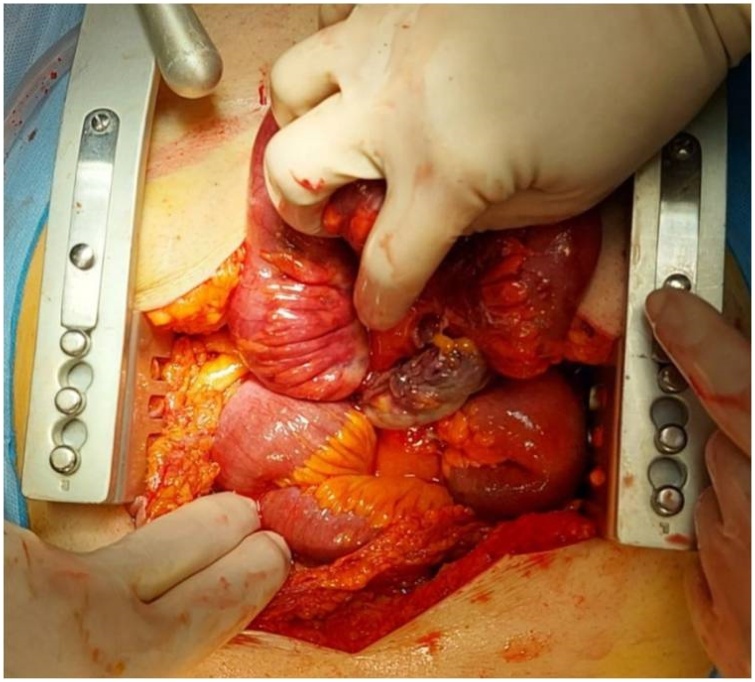
Necrotic annular Meckel’s diverticulum causing intestinal obstruction and ischemia: operative findings.

**Fig. 3:**
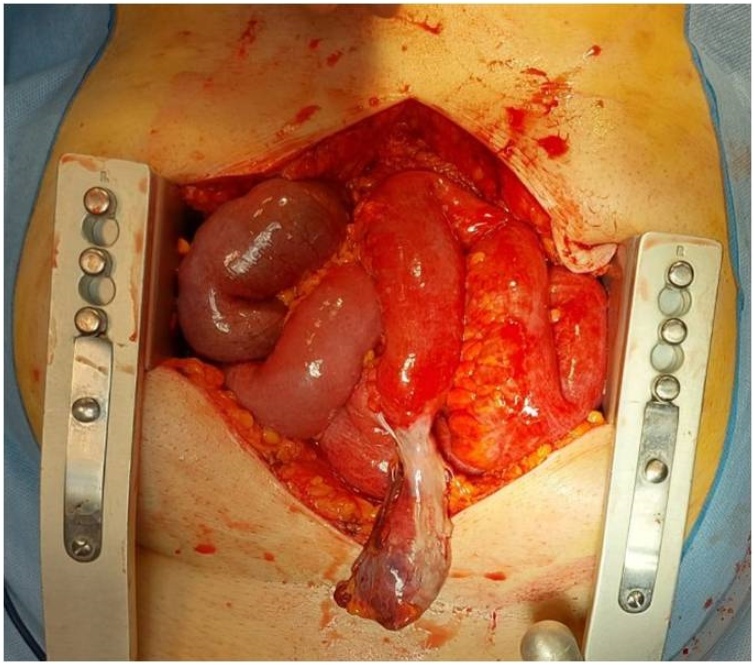
Necrotic annular Meckel’s diverticulum after lysis of the adhesion between its tip and base: operative findings.

**Fig. 4:**
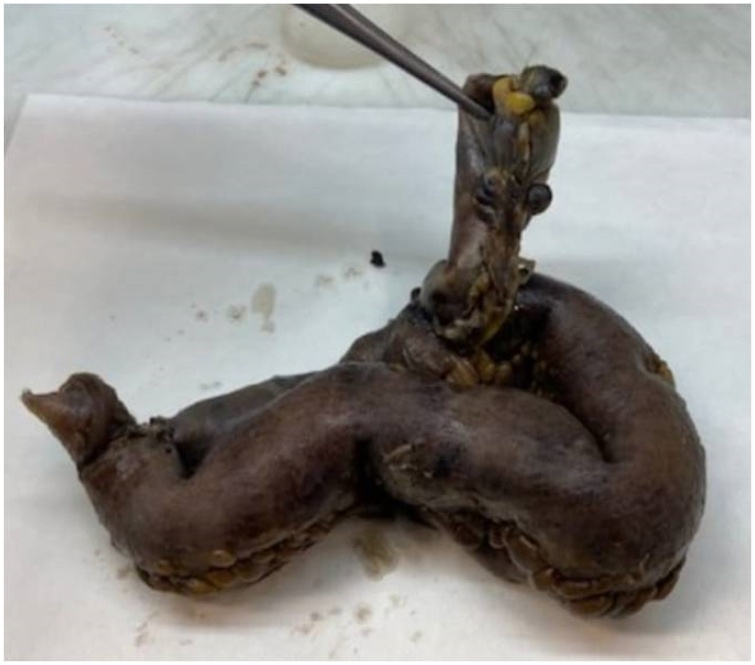
The surgical specimen fixed in formalin: 26 cm of terminal ileum bearing a Meckel’s diverticulum of 7.5 cm in length.

**Fig. 5:**
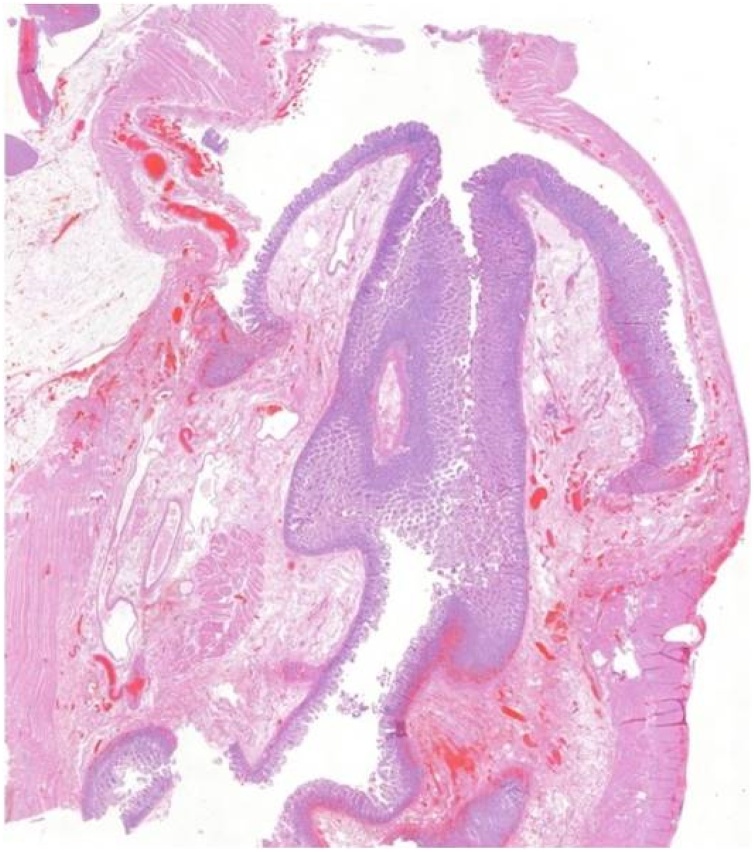
Photomicrograph section of necrotic Meckel’s diverticulum (haematoxylin and eosin, original magnification × 2).

A 70-year-old Caucasian male was admitted to the Emergency Department with a two-day history of spasmodic abdominal pain associated with inability to pass gas or stool, nausea and vomiting; vital signs were normal. The patient wasn’t taking any drug, referred habit on smoking but denied alcohol consumption. His past and familial medical histories were normal. He was retired from the work, married and of medium socio-economic status. Physical examination revealed abdominal distention, generalized abdominal pain at deep palpation without Blumberg’s sign. Laboratory tests reported high levels of glycemia (250 mg/dL), LDH (738 UI/L), C-reactive protein (266.5 mg/L) and neutrophilic leukocytosis (WBC 15.120 10^3^/μL). The patient was initially managed with fluids, intravenous broad-spectrum antibiotics and bowel rest. Abdominal contrast-enhanced computed tomography (CECT) showed small bowel obstruction caused by suspected MD (Fig. 1 A and B). The patient, after understanding the severity of his medical condition and accepting surgery, was taken emergently to the operating room by experienced general surgeons (the second and the last author) for exploratory laparotomy under general anesthesia. The patient was placed in the supine position on the operating table: intraoperatively a necrotic annular MD (located 50 cm proximal to the ileocecal valve) was found to strangulate part of the distal ileum, forming a constricting ring through an adhesion between its base and its tip and leading to intestinal obstruction and small bowel ischemia (Fig. 2). After lysis of the adhesion between the tip and the base of MD (Fig. 3), a segmental resection of the ischemic terminal ileum bearing the MD with latero-lateral mechanical ileoileal anastomosis was performed 40 cm distant from the competent ileocecal valve. Patient was given total parental nutrition for four days, an IV injection of Levofloxacin 500 mg once daily for five days and a SC injection of enoxaparin sodium 4.000 IU once daily for 21 days. The postoperative course was uneventful: abdominal drains were removed on the 7th postoperative day and postoperative laboratory tests were unremarkable. The patient was discharged on the 7th postoperative day in a stable condition and referred to Endocrinology Department for diabetes. The surgical specimen, fixed in formalin (Fig. 4), consisted of 26 cm of terminal ileum bearing a MD of 7.5 cm in length. Pathological examination showed the presence of inflammation and gangrene of MD (Fig. 5). The patient tolered the advice provided to avoid heavy lifting for four weeks and after a follow-up of six months is asymptomatic.

## 3 Discussion

This clinical case describes a extremely rare necrotic annular MD causing intestinal obstruction and small bowel ischemia. MD is the most common vitelline duct abnormality, found in approximately 2%–4% of the population in large autopsy and surgical series [4]. Generally MD ranges from 1–12 cm in length with a diameter of 0.3–7 cm and, when present, is located 7–200 cm proximal to the ileocecal valve [5]. The underlying genetic defects that cause MD have not been identified. The male-female ratio of MD is nearly equal in asymptomatic patients, but among symptomatic patients MD occurs more frequently in males (M:F ratio 3:1 to 4:1). MD mainly occurs in children being diagnosed mostly in the first 2 years of life [2] and rarely in adults over than 50 years. Clinical manifestation of MD arise from its complications. The overall incidence of complications due to MD ranges from 4%–16% [6]. Intestinal obstruction is the most common clinical presentation of MD in adults (24%–53%) [2]; other less common complications are lower gastrointestinal hemorrhage (25%–50%), tumors (0.5%–1.9%) and diverticulitis (20%) with or without perforation [7]. Symptomatic MD can represent a rare cause of mechanical obstruction of the small bowel. There are various mechanism by which MD can cause intestinal obstruction like a) volvulus of small bowel around a fibrous band extending from MD to umbilicus, b) intussusceptions of MD into the bowel lumen, c) incarceration of MD in hernia of the abdominal wall (Littre’s hernia), d) stricture secondary to chronic diverticulitis, e) MD lithiasis, f) tumor of MD, g) band extending between the diverticulum and the base of mesentery forming a loop in which a part of ileum may get stuck [8] and, as in our case, h) the presence of an annular MD, caused by an inflammatory adhesion between its tip and base, forming a ring in which a small part of ileum may be strangulated with ischemic necrosis of the intestinal wall. The patient affected by MD causing intestinal obstruction, as in our case, presents with symptoms and signs like as spasmodic abdominal pain, nausea, vomiting, inability to pass gas or stool and abdominal distention. Diagnosing MD may be a challenge: the vast majority are asymptomatic and typically undiagnosed or are only discovered during autopsy [9]. Different imaging studies (ultrasound, X-ray, angiography, contrast-enhanced computed tomography, Technetium-99 m pertechnetate scan, capsule endoscopy and magnetic resonance imaging) can be used for diagnosis but the sensitivity and specificity is low [5]. Radiological exams generally show complications of MD leading to surgery; direct observation of complicated MD during surgery will yield the correct diagnosis. In our case report CECT showed a suspected MD causing intestinal obstruction. The treatment of choice for the symptomatic MD is the surgical resection including diverticulectomy, wedge resection or segmental bowel resection depending on the integrity of diverticulum base and adjacent ileum as well the presence and location of ectopic tissue within MD [4,10]. The presence of ectopic tissue into MD cannot be accurately predicted intraoperatively by palpation or macroscopic appearance; however, when present, its location can be predicted based on height-to-diameter ratio. Long diverticula (height-to-diameter ratio >2) have ectopic tissue located at the body and tip requiring diverticulectomy; short diverticula (height-to-diameter ratio <2), having wide distribution of ectopic tissue include the base, require wedge or segmental bowel resection [10]. In our case report the presence of a necrotic MD with intestinal obstruction and ischemia required a segmental resection of the ischemic ileum bearing the MD. If the correct treatment of symptomatic MD is surgical resection, a controversy exists about silent MD concerning the prophylactic resection when MD is discovered during surgery because of possible complications following its resection. Some surgeons advise against prophylactic resection arguing that the morbidity is too high and that the reward is too low: in a systematic review Zani et al. [11] found a 5.3% risk of postoperative complications after prophylactic resection and a 1.3% risk of developing symptoms (without increasing late complications) after leaving MD in situ. However, among the patient series, a few compared resection of symptomatic MD to resection of silent MD and concluded that there are no discernible differences in the rates of morbidity and mortality [12]. Other authors claim that prophylactic resection of MD is recommended except in the face of contraindications like generalized peritonitis or others conditions that make resection more hazardous [13]. Still other surgeons choose a differentiated approach for silent MD, advocating for prophylactic resection upon meeting certain criteria that increase the likelihood of the silent MD becoming symptomatic. The largest of the retrospective patient series (The Mayo Clinic Experience with 1476 patients) identified 4 criteria which predispose to symptomatic MD: male sex, younger than 50 years, greater diverticular length than 2 cm and the presence of ectopic tissue; when meeting up to all of these criteria, 17%, 25%, 42% and 70% of Meckel diverticula were symptomatic [14]. In our case report only two criteria (male sex, greater diverticular length than 2 cm) predisposing to symptomatic MD were present.

## 4 Conclusion

MD represents the most common congenital anomaly of gastrointestinal tract. Diagnosis of MD is difficult due to its rarity and the absence of specific radiological findings and clinical presentation. Surgical resection represents the correct treatment of symptomatic MD.

## Declaration of Competing Interest

All the authors certify that there is no conflict of interest regarding the material discussed in the manuscript.

## Sources of funding

All the authors declare that this research didn’t receive any specific grant from funding agencies in the public, commercial, or not-for-profit sectors.

## Ethical approval

Ethical approval has been exempted by our institution because this is a case report and no new studies or new techniques were carried out.

## Consent

Written informed consent was obtained from the patient for publication of this case report and accompanying images. A copy of the written consent is available for the Editor-in-Chief of this journal on request.

## Author contribution

Giuseppe Evola: Drafting the manuscript and literature research.

Sebastiano Caramma: Operated on the patient, drafting the manuscript.

Giovambattista Caruso: Drafting the manuscript, literature research.

Riccardo Schillaci: Drafting the manuscript and literature research.

Carlo Reina: Drafting the manuscript and literature research.

Giuseppe Angelo Reina: Operated on the patient, revising the manuscript.

## Registration of research studies

Not applicable.

## Guarantor

The guarantor for this case report is Giuseppe Evola.

## Provenance and peer review

Not commissioned, externally peer-reviewed.
